# LCMV-mediated loss of virtual memory CD8 T cells yields a functionally enhanced T cell subset

**DOI:** 10.1016/j.isci.2025.113893

**Published:** 2025-10-28

**Authors:** Tabinda Hussain, Angela Nguyen, Daniel Thiele, Dulakara Kannangara, Zijian Huang, Ee Shan Pang, Alana Kirn, Sammy Bedoui, Kim L. Good-Jacobson, Meredith O’Keeffe, Kylie M. Quinn, Nicole L. La Gruta

**Affiliations:** 1 Immunity Program and Department of Biochemistry and Molecular Biology, Biomedicine Discovery Institute, Monash University, Clayton, VIC 3800, Australia; 2 Department of Microbiology and Immunology, University of Melbourne at the Peter Doherty Institute for Infection and Immunity, Melbourne, VIC 3000, Australia; 3 School of Health and Biomedical Sciences, Royal Melbourne Institute of Technology (RMIT) University, Bundoora, VIC 3082, Australia

**Keywords:** immunology, components of the immune system

## Abstract

The unique cytokine responsiveness of virtual memory T (T_VM_) cells endows them with a potent capacity for bystander activation and effector function. Here, we investigated the antigen-independent impact of microbial infections on T_VM_ cells. While *Salmonella typhimurium* or influenza A virus had no discernible effect, infection with lymphocytic choriomeningitis virus (LCMV) resulted in a rapid and profound depletion of T_VM_ and true naive (T_N_) cells. Unlike T_N_ cells, residual T_VM_ cells exhibited a less differentiated phenotype and heightened T cell receptor (TCR) responsiveness, compared to cells from uninfected mice. Notably, these changes persisted into advanced age, with sustained reductions in T_VM_ cell numbers and enhanced TCR sensitivity observed up to 18 months post infection, coincident with an attenuation of the senescent T_VM_ cell phenotype. These findings reveal a previously unrecognized mechanism by which early-life pathogen exposure imprints long term changes on T_VM_ cells, with broad implications for immune aging and lifelong immune competence.

## Highlights

• LCMV infection of mice causes a dramatic loss of T_VM_ cells
• The loss of T_VM_ cells selectively targeted the more differentiated or regulatory cells
• T_VM_ cells remaining after infection are more responsive to TCR mediated stimulation
• The loss, and coincident increase in TCR sensitivity, was maintained over the life course

## Graphical abstract

**Figure:**
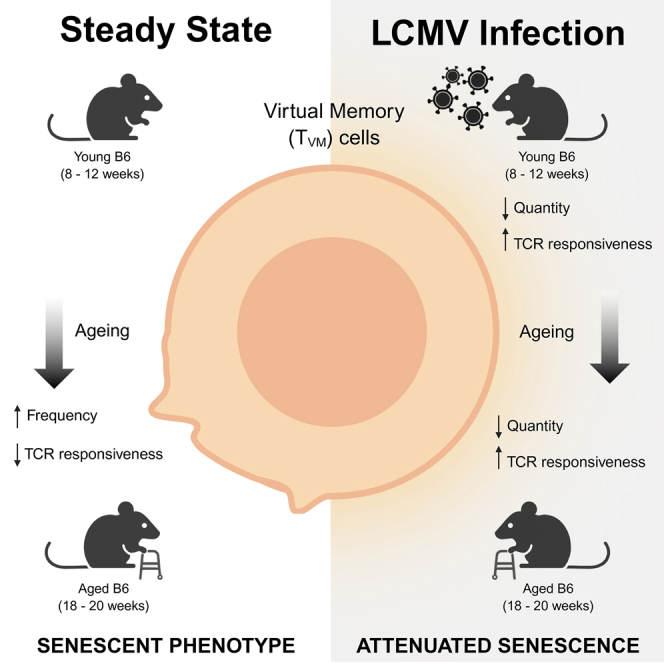

## Introduction

In the absence of acute antigen challenge, CD8 T cells in mice are predominantly comprised of three subsets: antigen-inexperienced naive T (T_N_) cells, antigen-experienced memory T (T_MEM_) cells, and a unique developmental CD8 T cell lineage of antigen-inexperienced T cells^1^^,^^2^ that are phenotypically and functionally similar to T_MEM_ cells. These cells are variously referred to as virtual memory T (T_VM_), memory phenotype, antigen-inexperienced memory T (T_AIM_), or innate memory T cells.^3^ T_VM_ cells have also been identified in humans,^3^^,^^4^^,^^5^^,^^6^^,^^7^ contained largely within the subset of T cells re-expressing CD45RA (T_EMRA_) and additionally expressing either NKG2A or KIRs. T_VM_ cells are of keen interest for a number of reasons, including their exquisite sensitivity to cytokines, such as type I IFNs, IL-15, and IL-12 shown to enhance T_VM_ cell’s innate-like functions,^5^^,^^6^^,^^8^ their ability to respond in an antigen-independent manner,^5^^,^^9^^,^^10^ and their associated bystander functions.^5^^,^^11^^,^^12^^,^^13^^,^^14^ Of particular relevance is the fact that the vast majority (>85%) of cells considered to be central memory T (T_CM_) cells (CD44^HI^CD62L^HI^) are T_VM_ cells, as indicated by low level expression of CD49d.^15^^,^^16^ T_VM_ cells have been implicated in responses to bacterial and viral infections,^12^^,^^13^^,^^17^^,^^18^ cancer immunosurveillance,^19^^,^^20^^,^^21^ and immunoregulation.^22^^,^^23^^,^^24^^,^^25^ They have also gained attention in the context of immune aging, as they show substantial accumulation, relative to T_N_ cells, in mice and humans (comprising up to 50% of all antigen-naive CD8 T cells^4^), and exhibit disproportionate age-related dysfunction in response to T cell receptor (TCR)-mediated stimulation, relative to T_N_ cells, both *in vitro*^4^ and *in vivo.*^26^ Mechanistically, T_VM_ cell dysfunction in aged mice can be attributed to senescence, with increased expression of γ-H2AX, Bcl-2, and p21, alongside increased basal mitogen-activated protein kinase phosphorylation, which is distinct from the exhausted phenotype acquired by conventional T_MEM_ cells.^4^ Notably, and distinct from T_MEM_ cells, T_VM_ cells largely retain their responsiveness to cytokines, in particular IL-15, in advanced age.^4^^,^^27^

Given their hallmark cytokine sensitivity, a major question that remains incompletely addressed is the extent to which pathogen exposure and its associated inflammation influence T_VM_ cell number and function in young and aged mice. Certainly, T_VM_ cells are not dependent on microbially derived cytokines as their number is unchanged in germ-free mice, which are raised in sterile conditions and contain no culturable gut flora.^8^^,^^28^ Helminth infection induces a transient cytokine (IL-15/IL-4)-mediated increase in T_VM_ cells that is not sustained into advanced age.^12^^,^^27^ Recently, T_AIM_ cells were found to be increased in feral mice and their F1 progeny, compared to SPF mice; however, cohousing SPF mice with feral mice, which induced alterations in bacterial colonization, did not change T_AIM_ cell frequency.^28^ Thus, the question of how non-helminth microbial exposure influences T_VM_ cells—both their heightened functionality in young adult mice and their senescence in aged mice—remains unresolved.

In this study, we explored the impact on T_VM_ cells of bacterial (*Salmonella typhimurium*) and viral (influenza A virus [IAV] and lymphocytic choriomeningitis virus [LCMV]) infections that are known to induce high levels of T_VM_-associated cytokines, including type I IFNs, IL-15, and IL-12.^5^^,^^6^^,^^8^^,^^9^^,^^10^^,^^29^^,^^30^^,^^31^^,^^32^^,^^33^^,^^34^ We found that only LCMV infection perturbed T_VM_ cell numbers, resulting in a permanent reduction in the frequency and number of T_VM_ cells. Moreover, the T_VM_ cells that remained after LCMV infection showed improved TCR-associated functionality and an attenuation of the senescent phenotype in advanced age. These findings represent a novel impact of infection on T_VM_ cells and support the notion that an individual’s response to infections across their lifespan, as well as the types of infection encountered, may contribute to both the heterogeneity in immune responses to infections observed across individuals as well as the varying kinetics of biological aging.

## Results

### Infection-driven changes to T_VM_ cells are pathogen-specific

*Salmonella enterica* (*S. enterica*) serovars, such as *S. typhimurium*, are known to induce IL-12 and IL-18 production in mice^29^^,^^30^ and IL-15 in humans,^31^^,^^32^ whereas IAV and, in particular, LCMV infection induces a strong type I IFN response and robust CD8 T cell activation.^33^^,^^34^^,^^35^ These pathogens exhibit distinct kinetics of infection and thus elicit distinct immune response kinetics. IAV (i.n. [intranasally]) and LCMV (i.v. [intravenously]) infections induce peak viral loads in the lungs and spleen, respectively, at ∼ day 3, with viral clearance by ∼8–12 days after infection.^36^^,^^37^^,^^38^^,^^39^ *S. typhimurium* (i.v.) on the other hand peaks in spleen and liver at ∼2 weeks and is cleared by 7–12 weeks after infection, with cytokines peaking at ∼3 weeks.^40^

**Figure 1:**
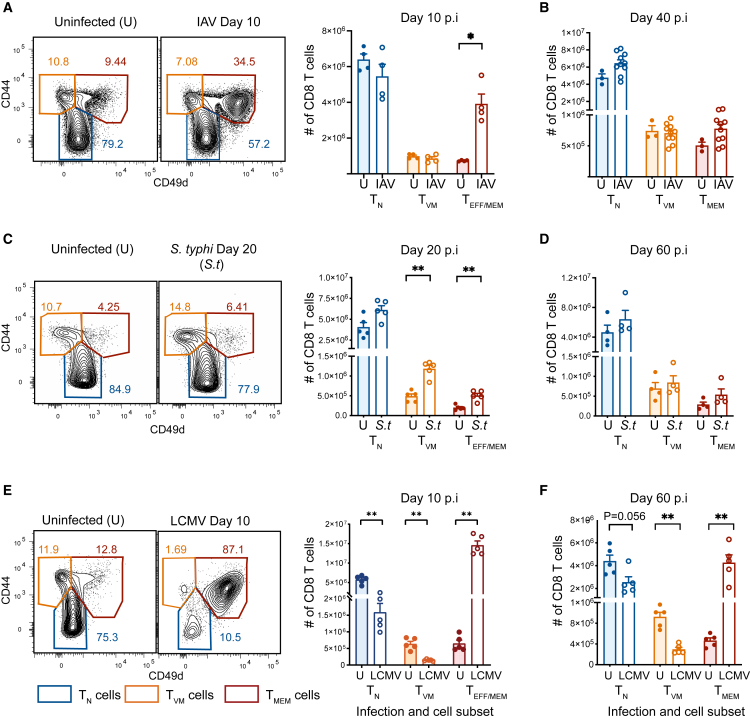
Impact of different infections on CD8 T cells at early and late timepoints B6 mice were either left uninfected or infected with IAV, *S. typhimurium*, or LCMV and splenocytes harvested at two timepoints to assess CD8 T cell subsets. (A) Representative contour plots gated on Live/TCR^+^/CD8^+^ T cells and depicting splenic T_N_, T_VM_, and T_MEM_ cells and bar graph depicting number of each subset at day 10 post-infection (p.i.) and at (B) days 40–60 p.i. of IAV infection (IAV) and uninfected (U) controls. (C) Representative contour plots gated on Live/TCR^+^/CD8^+^ T cells and depicting splenic T_N_, T_VM_, and T_MEM_ cells and bar graph depicting number of each subset at day 20 p.i. and (D) day 60 p.i. of *S. typhimurium* (*S.t*) infection and uninfected controls. (E) Representative contour plots gated on Live/TCR^+^/CD8^+^ T cells and depicting splenic T_N_, T_VM_ and T_MEM_ cells and bar graph depicting number of each subset at day 10 p.i. and (F) day 60 p.i. of LCMV-WE (LCMV) infection and uninfected controls. Data are representative of 2–3 experiments with *n* = 4–9 mice per group. ∗*p* < 0.05, ∗∗*p* < 0.01 using Mann-Whitney U test. Bar graphs represent mean ± SEM. See also Figures S1A, S1B, and S1E.

Mice were infected i.n. with the HKx31 strain of IAV. As expected, T_EFF_/_MEM_ cell numbers and proportions increased significantly at day 10 due to the antigen-specific CD8 T cell response (Figure 1A). However, T_VM_ cell number was unaffected by IAV, both at the acute stage of infection (day 10 p.i. [post-infection]) (Figure 1A) and in the memory phase after viral clearance (d40 p.i.) (Figure 1B), compared to uninfected mice. Thus, despite inducing a robust immune response, IAV infection did not influence T_VM_ cell numbers.

We next assessed the effect of a bacterial infection using an attenuated strain of *S. typhimurium* that causes a systemic infection that can be controlled in B6 mice.^40^^,^^41^ *S. typhimurium* induces a Th1 response, with increased production of IL-12 to facilitate bacterial clearance.^30^ It has previously been shown that T_VM_ cells respond to IL-12 stimulation with robust IFN-γ production.^42^^,^^43^ Splenic CD8 T cell subset number and proportions were measured at 20 and 60 days after i.v. infection with 200 CFU of *S. typhimurium* BRD509. In addition to the expected increase in T_EFF/MEM_ cells, T_VM_ cell numbers were also significantly and substantially (>2-fold) increased, compared to uninfected mice at day 20 p.i. (Figure 1C). This shift in T_VM_ cell numbers was transient; however, returning to baseline by day 60 (Figure 1D). These data suggest that the acute increase in inflammatory cytokines induced by *S. typhimurium* can drive a transient increase in T_VM_ cells; however, this is not maintained long term.

Finally, we examined the impact of LCMV infection on T_VM_ cells as it induces a strong CD8 T cell memory response and robust, acute, type I IFN responses,^33^ cytokines to which T_VM_ cells are particularly sensitive.^9^ Mice were infected with the acute LCMV-WE strain and splenic CD8 T cells assessed at day 10 and 60 after infection. Virus was largely cleared from serum and liver by day 14 after infection (Figure S1A).^39^ An acute strain of LCMV was used to assess shifts in T_VM_ cells that persisted beyond the duration of active viral replication and the presence of inflammatory cytokines. As expected, at day 10 after infection we observed a dramatic (∼25-fold) increase in T_EFF_ cells (Figure 1E), as a consequence of the large LCMV-specific CD8 T cell response,^35^ which dropped to a ∼10-fold difference at day 60, compared to uninfected mice (Figure 1F). Concurrently, we observed a dramatic (>60%) loss of both T_N_ cells and T_VM_ cells at day 10 after LCMV infection. Moreover, neither the T_VM_ cell nor T_N_ cell numbers were recovered at day 60 p.i. (Figure 1F), despite the fact that LCMV is cleared by day ∼10–14 post-infection.^39^ Thus, infection with acute LCMV results in a dramatic loss of T_N_ and T_VM_ cells in the acute phase following infection (day 10 p.i.), with this loss persisting long term (day 60 p.i.).

### LCMV infection rapidly depletes the antigen-naive CD8 T cell compartment

**Figure 2:**
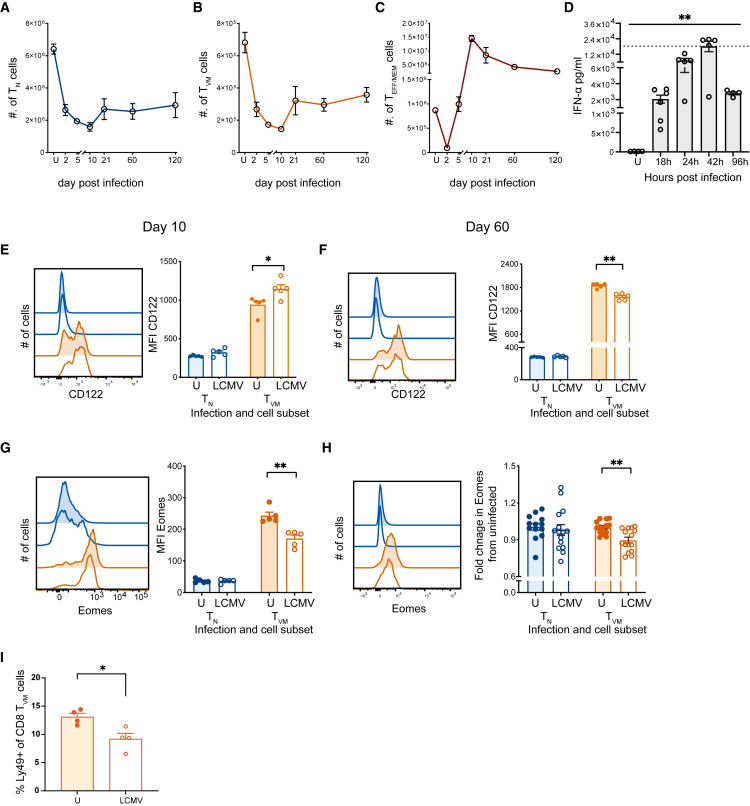
Kinetics of T cell loss and phenotype during LCMV infection B6 mice were infected with LCMV and early serum IFNα levels assessed, along with CD8 T cell number and phenotype assessed at day 10 and day 60. Number of splenic (A) T_N_, (B) T_VM_, and (C) T_EFF/MEM_ cells from day 2–120 p.i. of LCMV-WE infected and uninfected (U) controls. (D) Level of IFN⍺ in serum from 0 to 96 h p.i. Data (A, B, C, and D) is representative of 2–3 experiments with *n* = 4–5 mice per group, except day 21 and day 120 were analyzed once. ∗*p* < 0.05, ∗∗*p* < 0.01 using Mann-Whitney U test. Bar graphs represent mean ± SEM. Representative histograms showing CD122 expression and bar graph showing median fluorescence intensity (MFI) of CD122 staining on total T_N_ and T_VM_ cells at (E) day 10 and (F) day 60 after LCMV-WE infection and in uninfected (U) mice. Representative histograms showing Eomes expression and bar graph showing MFI of Eomes staining on total T_N_ and T_VM_ cells at (G) day 10 and (H) day 60 after LCMV infection and in uninfected (U) mice. (I) Percentage of T_VM_ cells expressing Ly49 in uninfected mice and at day 10 after LCMV-infection. Data (E–I) are representative 2–3 experiments with *n* = 4–5 mice per group. ∗*p* < 0.05, ∗∗*p* < 0.01 using Mann-Whitney U test. Bar graphs represent mean ± SEM. See also Figure S1E.

We next dissected the kinetics of the profound and prolonged CD8 T cell loss after infection. All CD8 T cell populations showed a marked, early (day 2 p.i.) drop to less than 50% of their original number (Figures 2A–2C). For T_N_ and T_VM_ cells, this loss continued with a nadir at day 10 of ∼30% of original number, followed by a modest recovery by day 21 and a relative stabilization thereafter at around 50% of their original numbers (Figures 2A and 2B). In contrast, T_EFF/MEM_ cells recover rapidly after their early loss, equaling pre-infection numbers by day 5 p.i. and then exceeding these numbers by >10-fold by day 10 p.i. (Figure 2C) due to the massive antigen-specific T cell expansion.^35^ Collectively, LCMV infection drives a rapid, profound, and sustained loss of both T_N_ and T_VM_ CD8 T cell populations.

LCMV infection drives a rapid type I IFN response that peaks early after infection.^33^ Indeed, type I IFN-mediated death of CD8 T cells in the first two days after LCMV infection has been documented previously,^44^^,^^45^^,^^46^^,^^47^ with blockade of type I IFNs rescuing CD8 T cells from apoptosis.^33^^,^^44^ While these studies suggested a preferential effect on T_MEM_ cells, here we show that it is the T_N_ and T_VM_ cells that are particularly affected. In agreement with previous studies, we observed significantly elevated serum levels of IFN-α within the first 24h of infection which peaked at 42h p.i. (Figure 2D), coincident with the precipitous drop in CD8 T cells (Figures 2A–2C). To verify that the loss of T_N_ and T_VM_ cells does not merely reflect migration to other tissues, we infected mice with LCMV and analyzed CD8 T cells from spleen, liver, and lungs at day 10 after infection. The reduction in T_N_ and T_VM_ cells was again evident in spleen, while T_VM_ numbers in liver and lungs were not increased (Figure S1B).^47^

T_VM_ cells are unique in their capacity to mediate both antigen-dependent and antigen-independent innate-like effector functions. We next assessed the expression of markers that reflect the unique functionality of T_VM_ cells, namely CD122 and Eomes, as T_VM_ cells show the highest expression of these markers and require the expression for development.^1^^,^^9^^,^^10^^,^^15^ At 10 days after infection T_VM_ cells showed a significant increase in CD122, as has been observed following exposure to IL-15^27^^,^^48^ (Figure 2E). In contrast, at a later time point (day 60), T_VM_ cells showed a significant drop in CD122 expression, suggesting that the LCMV-mediated loss of T_VM_ cells had selectively targeted CD122^hi^ cells (Figure 2F). Concordant with this, we also observed a selective reduction of Eomes^hi^ cells at both day 10 and day 60 (Figures 2G and 2H). Enhanced reactivity to cytokines in T_VM_ cells has been associated with increased Eomes expression.^49^ The T_VM_ cell population is relatively heterogeneous and includes a population of Ly49+ cells that have been shown to exhibit regulatory properties.^25^^,^^50^^,^^51^ We found that LCMV infection also resulted in a selective loss of this regulatory subset (Figure 2I). Thus, it appears that LCMV-mediated depletion of T_VM_ cells may selectively target the more innate-like, cytokine-sensitive subset as well as T_VM_ cells with regulatory capacity.

### LCMV infection of young mice results in improved T_VM_ cell responses to TCR-mediated stimuli

**Figure 3:**
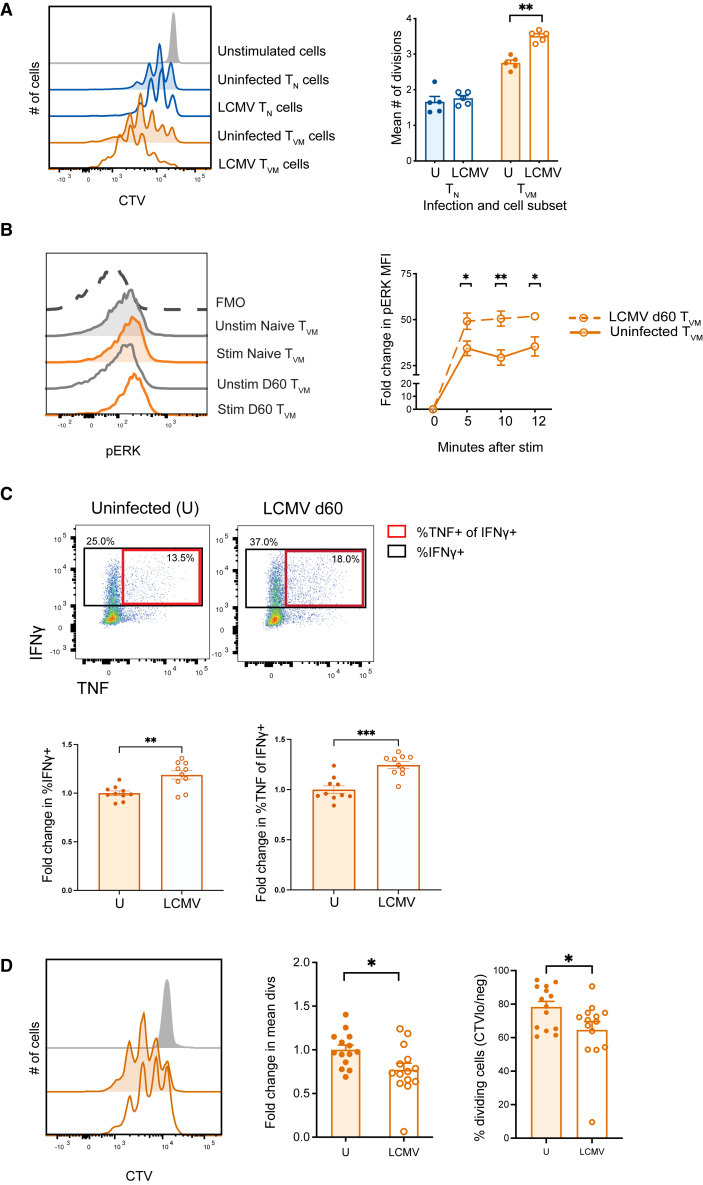
LCMV infection results in a functionally superior T_VM_ subset B6 mice were infected with LCMV and various measures of CD8 T cell function assessed at day 60 after infection. (A) Representative histograms depicting CTV dilution of sorted T_N_ and T_VM_ cells after 65 h of polyclonal stimulation (anti-CD3/8/11a) and bar graph showing the corresponding mean divisions. (B) Representative histograms showing *p*-ERK expression in sorted T_VM_ cells from uninfected and day 60 LCMV infected mice at 8 min after stimulation and change in *p*-ERK expression relative to unstimulated controls from 0 to 12 min of polyclonal (anti-CD3/CD8/CD28 Ab) stimulation. (C) Representative plots and summary data indicating proportion of sorted T_VM_ cells producing IFNɣ (black box) or IFNɣ and TNF (red box) after 36 h of polyclonal stimulation (anti-CD3/CD8/CD11a Ab). Fold changes calculated relative to average %IFNɣ+ cells or average %TNF+ of IFNɣ+ cells in T_VM_ cells from uninfected (U) mice. (D) Representative histograms depicting CTV dilution of sorted T_VM_ cells after 5days stimulation with 100 ng of cIL-15 and bar graph showing change in mean divisions relative to average divisions in uninfected T_VM_ cells, with data pooled from two different experiments. Data are representative of 2–3 experiments or combined from 2 independent experiments (B and D) with *n =* 4–5 mice per group. ∗*p* < 0.05, ∗∗*p* < 0.01 using Mann-Whitney U test. Bar graphs represent mean ± SEM. See also Figures S1C, S1E, and S1F.

Given the dramatic impact of LCMV on CD8 T_N_ and T_VM_ cell numbers (Figures 2A and 2B), we next assessed the impact of LCMV infection on the functionality of the remaining T_N_ and T_VM_ cells in response to both TCR-mediated and innate stimuli. These functional analyses were performed on CD8 T_N_ and T_VM_ cell subsets from mice that had been infected with LCMV >2 months earlier (LCMV day 60) and compared to uninfected mice. First, the proliferative capacity of T_N_ and T_VM_ cells was determined by CellTrace Violet (CTV) dilution in response to *in vitro* TCR stimulation (Figure 3A). Intriguingly, while the mean number of divisions was unchanged in T_N_ cells from infected compared to uninfected mice, T_VM_ cells from infected mice showed a significantly increased proliferative capacity compared to uninfected controls (Figure 3A). In addition, T_VM_ cells from infected mice showed enhanced phosphorylation of ERK (pERK) after TCR stimulation (Figure 3B), compared to uninfected mice. These data indicate that, while LCMV mediates depletion of cytokine-responsive T_VM_ cells, the remaining T_VM_ cells exhibit enhanced responsiveness to TCR-mediated stimulation.

T cell polyfunctionality, or the ability to produce multiple cytokines and/or effector molecules, is associated with increased cytokine production on a per cell basis and directly augments viral and bacterial clearance.^52^^,^^53^^,^^54^ CD8 T cells typically produce IFNγ and TNF hierarchically, with all responding T cells making IFNγ, and a subset of those also making TNF.^54^ The fraction of T_VM_ cells able to make IFNγ was significantly increased after LCMV infection (Figures 3C and S1C). Moreover, there was a significant increase in the proportion of IFNγ-producing T_VM_ cells that also produced TNF from infected mice compared to uninfected mice (Figures 3C and S1C).

A hallmark of T_VM_ cells is their sensitivity to cytokines, in particular IL-15,^3^^,^^10^^,^^15^^,^^27^ a phenotype that is largely retained in aged mice.^4^ We assessed the proliferative capacity of T_N_ and T_VM_ cells from uninfected and LCMV-infected (day 60 p.i.) mice in response to stimulation with IL-15. Sorted T_VM_ cells were cultured in the presence of IL-15/IL-15Rα complexes (cIL-15),^5^^,^^55^ and proliferation was assessed after 5 days. In contrast to the significantly increased sensitivity to TCR-mediated stimulation, T_VM_ cells from LCMV-infected mice showed reduced proliferation after *in vitro* IL-15 stimulation (Figure 3D). Together, these results suggest that the massive inflammation driven by LCMV infection may result in the selective loss of T_VM_ cells with more pronounced bystander activity (higher cytokine sensitivity, lower TCR sensitivity) leaving a population of T_VM_ cells enriched in adaptive function (higher TCR sensitivity).

### T_VM_ cell number is not recovered with aging in previously LCMV-infected mice

Work by ourselves and others has shown that, following helminth infection, there is an acute increase in the number of T_VM_ cells; however, this increase was transient and not maintained for extended periods post infection^12^^,^^18^^,^^27^ or into advanced age. To determine whether the impact of LCMV infection on T_VM_ cell number and function could persist over the entire lifetime, 8–12 week-old mice were infected with LCMV and then aged to 18–19 months of age (hereafter referred to as LCMV_AGED_ mice).

**Figure 4:**
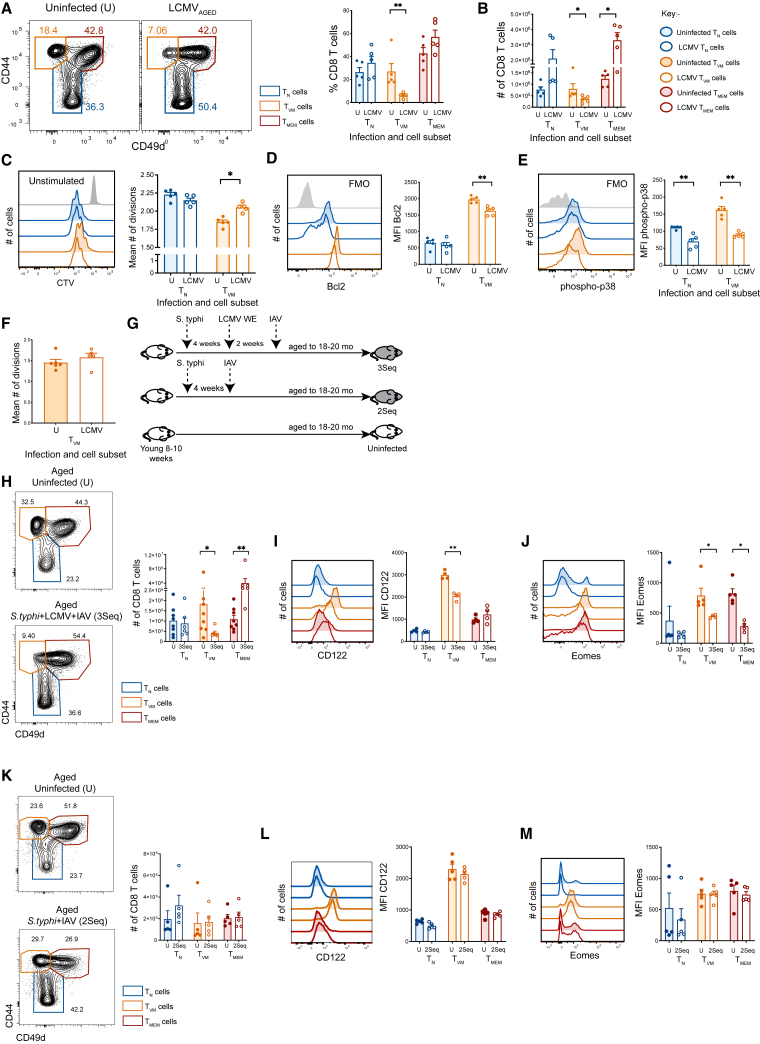
T_VM_ cells from LCMV_AGED_ mice maintain the quantitative and qualitative changes over a life course irrespective of additional infections Young B6 mice were infected with LCMV ± *S. typhimurium* and IAV, and then aged to 18–20 months. (A) Representative contour plots gated on Live/TCR^+^/CD8^+^ T cells and depicting T_N_, T_VM_, and T_MEM_ cells from LCMV-infected and then aged (LCMV_AGED_) and uninfected (U) aged mice (left) and bar graph depicting frequency (right) and (B) number of cells in each subset. (C) Representative histograms depicting CTV dilution of sorted T_N_ and T_VM_ from aged, previously infected (LCMV) and aged uninfected (U) mice after 65 h of polyclonal stimulation (anti-CD3/8/11a) (left) and bar graph showing the corresponding mean divisions (right). (D) Representative histograms showing Bcl-2 expression in T_N_ and T_VM_ cells from aged, previously infected (LCMV) and aged uninfected (U) mice (left) and bar graph (right) showing MFI of Bcl-2 on all T_N_ and T_VM_ cells. (E) Representative histograms showing basal phosphorylated (p)-p38 levels in sorted T_N_ and T_VM_ cells and bar graph showing MFI of p-p38 staining for all T_N_ and T_VM_ cells from aged, previously infected (LCMV) and aged uninfected (U) mice. (F) Mean divisions of sorted T_VM_ cells after 5 days of *in vitro* stimulation with 50 ng cIL-15 from previously LCMV-infected and aged (LCMV) and aged uninfected (U) mice. (G) Schematic depicting sequential infection of mice with 3 (*S. typhimurium*, LCMV, IAV; “3Seq”) or 2 pathogens (*S. typhimurium* and IAV; “2Seq”) and then aged. (H) Representative contour plots gated on Live/TCR^+^/CD8^+^ T cells and depicting T_N_, T_VM_, and T_MEM_ cells from *S. typhimurium*, LCMV and then IAV infected and then aged (3Seq) and uninfected (U) mice and bar graph depicting number of cells in each subset. (I) Representative histograms depicting expression of CD122 on T_N_ and T_VM_ cells and the MFI of CD122 staining on all cells in each subset. (J) Representative histograms depicting Eomes expression and the respective MFI of Eomes staining on all T_N_ and T_VM_ cells from 3Seq and uninfected (U) mice. (K) Representative contour plots gated on Live/TCR^+^/CD8^+^ T cells and depicting T_N_, T_VM_ and T_MEM_ cells from 2Seq and uninfected (U) mice and bar graph depicting number of cells in each subset. (L) Representative histograms depicting expression of CD122 on T_N_ and T_VM_ cells and MFI of CD122 staining on all cells in each subset. (O) Representative histograms depicting Eomes expression and the respective MFI of Eomes staining on all T_N_ and T_VM_ cells from 2Seq and uninfected mice. (A, B, C, D, E, and F) Data represent 3–4 experiments with *n =* 4–5 mice per group. ∗*p* < 0.05, ∗∗*p* < 0.01 using Mann-Whitney U test. Bar graphs represent mean ± SEM. (H–M) Data are representative of 2–3 experiments with *n =* 3–5 mice per group, ∗*p* < 0.05, ∗∗*p* < 0.01 using Mann-Whitney U test. Bar graphs represent mean ± SEM. See also Figure S1D.

Remarkably, the loss of T_VM_ cells observed early after infection (Figure 2B) was maintained out to 18–19 months of age (Figures 4A, 4B, and S1D), with the T_VM_ cell population in LCMV_AGED_ mice representing approximately half of the number of T_VM_ cells in uninfected aged mice (Figure 4B). Notably, the long-term reduction in CD8 T cell numbers was only observed for T_VM_, and not for T_N_, cells in aged mice. As previously described, T_MEM_ cells that had responded to LCMV infection remained elevated in aged mice (Figure 4B). Importantly, to ensure that T_MEM_ cells were not downregulating CD49d, and thus being misclassified as T_VM_ cells in aged mice, we assessed CD122 expression—which is commonly used in place of CD49d to identify T_VM_ cells—on T_N_, T_MEM_, and T_VM_ cells from aged naive and infected mice. The cells identified as T_VM_ cells showed high expression of CD122, which was distinct from the lower CD122 expression observed on T_MEM_ cells (Figure S2), suggesting that the CD49dlo cells in aged mice are true T_VM_ cells. Collectively, these data indicate that LCMV infection in early life results in permanent quantitative changes to the T_VM_ cell population.

### T_VM_ cells from LCMV-infected and aged mice show heightened TCR responsiveness

An established characteristic of T_VM_ cells is their development of a senescent phenotype with age, which is uncharacteristic of antigen-naive T cells, and incorporates the acquisition of deficits in TCR-mediated proliferation, as well as other markers of senescence such as elevated levels of Bcl-2 and basal phosphorylation of p38.^4^

Given the marked alteration in T_VM_ cell number and phenotype with prior LCMV infection, which is sustained through the life course, we explored whether the functional changes in T_VM_ cells—namely their heightened capacity to respond to TCR stimuli—was also maintained. T_N_ and T_VM_ cells from LCMV_AGED_ mice were assessed for responsiveness to TCR and IL-15 stimulation. Notably, T_VM_ cells from LCMV_AGED_ mice showed a significant increase in TCR-driven proliferation compared to uninfected aged mice (Figure 4C), suggesting that this functional advantage, acquired during early-life exposure to LCMV, was maintained over the life course. Intriguingly, Bcl-2 and basal phosphorylation of p38 in LCMV_AGED_ mice was significantly reduced compared to uninfected aged controls (Figures 4D and 4E), reflecting an attenuation of the senescent phenotype. We observed no difference in responsiveness to IL-15 by T_VM_ cells from LCMV_AGED_ mice compared to uninfected aged controls (Figure 4F). Collectively, these data indicate that LCMV infection permanently alters the number and function of T_VM_ cells such that they retain substantive TCR responsiveness in advanced age, possibly by purging the T_VM_ repertoire of cytokine-sensitive cells that are more likely to become senescent in advanced age.

### Additional infections do not alter T_VM_ cell loss induced by LCMV infection

We have shown that early life exposure to LCMV infection drives a reduction in T_VM_ cells and a change in their function, which is sustained over a life course. To determine whether these LCMV-related changes in T_VM_ cell number are mitigated or modulated by additional infections that drive a strong inflammatory environment, we sequentially infected young adult mice with *S. typhimurium*, LCMV, and IAV (3Seq model) and analyzed T cells at 18–19 months of age (Figure 4G). *S. typhimurium* is known to induce a robust IL-15 response^31^^,^^56^^,^^57^ and results in a modest and transient increase in T_VM_ cells in mice (Figures 1C and 1D). The sequence of infections (*S. typhimurium*>LCMV>IAV) was designed to determine whether LCMV could still effectively deplete T_VM_ cells in this setting and whether such depletion would persist following a subsequent challenge with IAV. Intriguingly, we found that LCMV, even in the context of other proinflammatory infections, was able to cause a severe (∼3-fold) and prolonged depletion of T_VM_ cells (Figure 4H). This loss of T_VM_ cells and the associated loss of CD122 and Eomes expression (as seen at day 60 after LCMV) (Figures 4I, 4J, and 2E–2H) were not seen in mice infected with only *S. typhimurium* and IAV (2Seq model) and then aged out to 18–20 months (Figures 4K–4M), as expected, based on infections with individual pathogens (Figures 1A–1D). Collectively, these data demonstrate that the long term numeric, phenotypic, and functional changes in T_VM_ cells are pathogen-specific, and that LCMV infection has a dominant impact on T_VM_ cells that cannot be modulated by additional infection-driven inflammation.

## Discussion

We have demonstrated that LCMV infection can significantly impact T_VM_ cells both quantitatively and qualitatively. These changes manifest as a sustained reduction in cell numbers, with the remaining T_VM_ cells showing improved TCR-mediated functionality. Importantly, these changes seem to be permanent, remaining long after initial infection, and strikingly, even into old age, ultimately attenuating the dysfunction observed with aging.^4^

Additionally, we show through sequential infection models that the age-associated accumulation of T_VM_ cells is not enhanced with a history of pathogen exposure. We found that even infections like *Salmonella*, which can induce substantial levels of IL-12 and IL-15,^29^^,^^30^^,^^31^^,^^56^^,^^57^ a cytokine signature that favors T_VM_ cells^5^^,^^8^^,^^10^^,^^15^ do not lead to a major expansion in the T_VM_ cell population. Moreover, we observed that varied pathogen exposure does not impart a different phenotype or functionality in T_VM_ cells and that LCMV infection, uniquely, causes a major and permanent quantitative shift in T_VM_ cells. We propose that this shift is caused by the selective death of a subset of T_VM_ cells due to excessive exposure to a high type I IFN milieu; however, further studies are required to define the precise mechanism by which LCMV drives T_VM_ cell loss.

We found that T_VM_ cells, along with other CD8 T cells, underwent attrition during early LCMV infection, coincident with an exaggerated type I IFN response. This type I IFN-induced loss of T cells following LCMV and *Listeria monocytogenes* has been described previously.^44^^,^^58^ Indeed, blockade or absence of type I IFN signaling can lead to better outcomes during LCMV infection,^33^^,^^44^ as well as during *L. monocytogenes* and malarial infections.^58^^,^^59^ Indeed, multiple type I IFN-inducing infections in humans are characterized by a very early and severe lymphopenia, including *L. monocytogenes*, Lassa fever, Ebola, and SARS coronavirus,^44^^,^^46^^,^^60^^,^^61^^,^^62^ indicating that the T_VM_ phenotype we observe is likely not restricted to LCMV. It has been suggested that this infection-induced T cell depletion may be a mechanism to favor the development of new T cell responses or enhance TCR diversity in the recall response.^46^ Here, we reveal another possible mechanism by which this depletion may drive improved responses to antigen: by augmenting the TCR responsiveness of the remaining T_VM_ cells. One interpretation of our results could be that T_VM_ cells represent a functionally heterogenous population, and that LCMV infection selectively targets T_VM_ cells exhibiting greater cytokine sensitivity, leaving a less-differentiated subset behind, and thus “purging” cells which may have a greater potential to become senescent over time, due to long-term cytokine stimulation in the increasingly inflammatory (inflammaged) aged environment. Alternatively, the selective loss of T_VM_ cells with a regulatory function (Ly49+) may also augment the responsiveness of the remaining T cells. While we have no direct evidence for these hypotheses, heterogeneity in the T_VM_ cell population has been described before in both mice and humans, with reports showing differential capacity to respond to cytokines or to mediate effector-like responses.^7^^,^^49^^,^^63^^,^^64^ Of particular note, Smith and colleagues used timestamped mice to track the development and survival of T_VM_ cells. They found that those generated early in life exhibited a more “effector”-like population expressing markers of innate functionality including Eomes, CD122, and NK cell receptors and were more responsive to inflammation.^49^ These findings have implications for understanding how age-associated senescence of CD8 T cells is regulated and how aged CD8 T cell responses can be improved in subsequent immune responses.

T_VM_ cells have rapid TCR-mediated proliferation and cytokine-driven responses in young mice; however, aging results in a loss of TCR functionality and a senescent phenotype, without loss of cytokine responsiveness. In this study, we showed that the improved TCR-mediated responses in T_VM_ cells from LCMV-infected mice were coincident with reduced sensitivity to IL-15 stimulation. Similar observations of an inverse relationship between TCR signaling and bystander activation have been made previously, where activation of bystander cytotoxicity via NKG2D, in CD8 T cells from human and mice, was associated with impaired TCR signaling. This was mediated by inhibitory complexes formed by sestrins, which localize with NKG2D in the cytosol and disrupt activation of TCR signaling complex.^65^

While earlier studies have reported that LCMV-mediated attrition of T_N_ cells is not as severe as for CD44^HI^ memory phenotype T cells,^44^ we observed a similar reduction in both T_N_ cells and T_VM_ cells as early as day 2 p.i. The significant (>2-fold) reduction in T_N_ and T_VM_ cells after LCMV infection, which is maintained long term, reduces the antigen-naive CD8 T cell pool down to the level of attrition that is observed in old age. However, we did not observe a further reduction in T_N_ cells with aging in LCMV_AGED_ mice, and indeed there was a trend toward increased T_N_ cells in LCMV_AGED_ mice compared to uninfected mice. A tempting inference here would be that the T_N_ cell subset that survives after LCMV infection is better able to survive the age-induced immune environment. A rapid reduction in naive CD8 T cells has also been observed in HIV infection,^66^^,^^67^^,^^68^ which is known to induce an “immune-ageing” phenotype marked by an increase in terminally differentiated CD8 T cells.^66^^,^^69^^,^^70^ It would therefore be pertinent to explore whether other viral infections in humans, which induce attrition in the T cell compartment, can also impart similar qualitative changes in antigen-naive T cell compartments.

Collectively, our study shows that early life pathogen exposure can permanently alter the quantity and quality of T_VM_ cells in a pathogen-specific manner. Moreover, this work suggests that the age-associated defect in T_VM_ cells is not absolute and may be at least partially attenuated by early life pathogen exposure. These findings may in part explain the heterogeneity in immune aging and may be leveraged to retain functionality and improve antigen-specific responses in advanced age.

### Limitations of the study

This study describes the observation that LCMV infection selectively depletes T_N_ and T_VM_ cells over the long term, resulting in a population of T_VM_ cells that show superior responsiveness to TCR-mediated stimuli *in vitro*. While type I IFNs have previously been demonstrated to be responsible for CD8 T cell loss after LCMV infection,^44^^,^^45^^,^^46^^,^^47^ our study does not detail the mechanisms by which this yields a functionally augmented population. Moreover, due to the difficulty in studying T_VM_ cells in TCR transgenic mouse models, this study does not demonstrate augmentation of *in vivo* TCR responsiveness. Although a number of Type I IFN-inducing infections have been found to induce a similar early, robust depletion of CD8 T cells,^44^^,^^60^^,^^61^^,^^62^^,^^66^^,^^67^ we have not generalized our findings to these other infection models. Finally, while LCMV-mediated depletion of CD8 T cells has been shown in both male and female mice, we have not confirmed our specific observations in male mice.

## Resource availability

### Lead contact

Further information and any requests should be directed to and will be fulfilled by the lead contact, Professor Nicole L. La Gruta (nicole.la.gruta@monash.edu).

### Materials availability

No new materials were generated by this study.

### Data and code availability

This paper does not report any original code and does not contain large-scale omics datasets. Any additional information or raw data required to reanalyze the data reported in this study are available from the lead contact upon request.

## STAR★Methods

### Key resources table

**Table:** 

REAGENT or RESOURCE	SOURCE	IDENTIFIER
**Antibodies**		

Anti-CD8a-BUV395 (clone 53-6.7)	BD Biosciences	Cat#563786; RRID: AB_2732919
Anti-CD44-PE-Cy7 (clone IM7)	BD Biosciences	Cat#25-0441-82; RRID: AB_1727484
Anti-CD44-FITC (clone IM7)	BD Biosciences	Cat#553133; RRID: AB_2076224
Anti-TCRb-AF700 (clone H57-597)	Biolegend	Cat#109224; RRID: AB_1027648
Anti-TCRb-BV421 (clone H57-597)	Biolegend	Cat#109229; RRID: AB_10933263
Anti-CD49d-AF647 (clone R1-2)	Biolegend	Cat#103614; RRID: AB_528837
Anti-CD49d-PE-Cy7 (clone R1-2)	Biolegend	Cat#103618; RRID: AB_2563700
Anti-CD122-FITC (clone TM-b1)	eBioscience	Cat#11-1222-82; RRID: AB_465189
Anti-CD122-BV421 (clone TM-b1)	BD Biosciences	Cat#566301; RRID: AB_2917942
Anti-CD122-PE (clone TM-b1)	Biolegend	Cat#123210; RRID: AB_940617
Anti-CD5-PerCP-Vio700 (clone 53-7.3)	Miltenyi Biotec	Cat#130-103-866; RRID: AB_2658613
Anti-Ly49C/I/F/H-FITC (clone 14B11)	Invitrogen	Cat#11-5991-85; RRID: AB_465341
Anti-Bcl2-FITC (clone 10C4)	eBioscience	Cat#11-6992-42; RRID: AB_10734060
Anti-Eomes-PE-Cy7 (clone Dan11Mag)	eBioscience	Cat#25-4875-82; RRID: AB_2573454
Anti-Phospho-p38 MAPK (Thr180/Tyr182) (clone D3F9)	Cell Signaling Technology	Cat#4511T; RRID: AB_2139682
Anti-Phospho-p44/42 MAPK (ERK1/2) (Thr202/Tyr204) (clone D13.14.4E)	Cell Signaling Technology	Cat#4370S; RRID: AB_2315112
Anti-TNF-PE (clone MP6-XT22)	Biolegend	Cat#12-7321-81; RRID: AB_466198
Anti-IFNg-FITC (XMG1.2)	BD Biosciences	Cat#554411; RRID: AB_395375
Anti-Rabbit IgG F(ab’)2 fragment	Cell Signaling Technology	Cat#8885; RRID: AB_2797677
Anti-VL-4 Ab	Prepared in-house	Battegay et al.^71^
Peroxidase-conjugated AffiniPure Goat Anti-Rat IgG (H + L)	Jackson ImmunoResearch	Cat#112-035-003; RRID: AB_2338128

**Bacterial and virus strains**		

Lymphocytic choriomeningitis virus – WE strain (acute)	Prepared in-house	Cooper et al.^39^
Lymphocytic choriomeningitis virus – Docile strain (chronic)	Prepared in-house	Cooper et al.^39^
*Salmonella* Typhimurium - BRD509 strain (ΔaroA ΔaroD mutant of the SL1344 strain)	Prepared in-house	Turner et al.^72^
Influenza A virus A/Hong Kong/X31 (HKx31) H3N2	Prepared in-house	Mintern et al.^37^

**Chemicals, peptides, and recombinant proteins**		

Cell Trace Violet	Invitrogen	Cat#C34557
Fixable viability stain 700	BD Biosciences	Cat#564997
Live/Dead Fixable Near IR	Invitrogen	Cat#L34975
Live/Dead Fixable AquaBlue	Invitrogen	Cat#L34966
Propidium Iodide	BD Biosciences	Cat#556463
Percoll	Sigma Aldrich	Cat#P4937
Red blood cell lysis buffer	Sigma Aldrich	Cat# 11814389001

**Critical commercial assays**		

LEGENDplex MU Anti-Virus Response Panel (13-plex)	Biolegend	Cat#740621
Cytofix/Cytoperm Fixation/Permeabilization Kit	BD Biosciences	Cat#554714
Phosflow Lyse/Fix Buffer 5X	BD Biosciences	Cat#558049
Phosflow Perm Buffer II	BD Biosciences	Cat#558052
Foxp3/Transcription Factor Staining Buffer Set	eBioscience	Cat#00-5523-00

**Experimental models: Organisms/strains**		

Inbred C57BL6/J mouse strain	Bred in-house	–

**Other**		

Accucheck counting Beads	Invitrogen	Cat#PCB100

### Experimental model and study participant details

Female C57BL/6 mice were bred and housed at Monash Animal Research Platform (MARP) or at the University of Melbourne in specific pathogen-free (SPF) conditions. Young mice used in this study were defined as 8–12 weeks old and aged mice were defined as 18–20 months old. Experimental procedures were conducted with prior approval from the Monash University Animal Ethics Committee (AEC18886 and AEC17866).

### Method details

#### Infections

For influenza A virus (IAV) infections, mice were anesthetized by isoflurane inhalation and intranasally infected with 1x10^4^ plaque-forming units (pfu) of A/Hong Kong/X31 (HKx31) H3N2 strain in 30 μL dPBS.^54^ For *S.* Typhimurium infections, the BRD509 strain of *S.* Typhimurium was used (ΔaroA ΔaroD mutant of the SL1344 strain).^72^ Mice were briefly warmed in their cages before administering 200 colony-forming units (cfu) of BRD509 intravenously via the tail vein in 100 μL dPBS. For lymphocytic choriomeningitis virus (LCMV) infections, mice were infected intravenously (i.v.) with either 3000 PFU of LCMV-WE to induce an acute LCMV infection, or 2 × 10^6^ PFU of LCMV-Docile to induce a chronic LCMV infection.^39^ Mice were briefly warmed in their cages before administering 3000 pfu of LCMV-WE intravenously via the tail vein in 100 μL dPBS. For sequential infection model involving two pathogens (2Seq), young mice were first infected with *S.* Typhimurium, then, following four weeks, mice were infected with HKx31. For sequential infection model involving three pathogens (3Seq), mice were first infected with *S.* Typhimurium, then four weeks later, infected with LCMV-WE, then, two weeks later, infected with HKx31. Mice in the 2Seq and 3Seq infection models were then aged until 18–20 months prior to analysis. All uninfected control mice were age-matched to within 1 week of their infected counterparts for all experiments up to day 120. All uninfected control mice in aging experiments were age-matched to within 4 weeks of their infected counterparts, with all being 18–20 months old.

#### Focus formation assay

The right lobe of the liver was collected, and DMEM (Gibco) with 2% FCS was added to obtain 500 mg/mL of liver suspension. One stainless steel bead (Qiagen) was added, and liver tissues were homogenized for 5 min at 50 oscillations/second using the TissueLyser LT (Qiagen). Samples were centrifuged at 15,000 RPM for 10 min, and the supernatant harvested. The focus forming assay was performed as described.^73^ Briefly, liver homogenate or sera was serially diluted in DMEM with 2% FCS, and added to cultured MC57G cells. Samples were incubated at 37°C for 4 h, and an overlay mixture comprised of 2% methylcellulose (Sigma Aldrich) and 2xDMEM (Gibco) added. Samples were incubated at 37°C for 48 h to allow for focus formation. To detect foci, cells were fixed with 4% formaldehyde (Sigma-Aldrich) in PBS, and stained sequentially with anti-VL-4 antibody^71^ and goat anti-rat IgG (Jackson ImmunoResearch). Foci spots were visualised using the Vector HRP substrate kit, and counted to quantify viral load.

#### Serum cytokine analysis

Whole blood was collected via cardiac puncture. Following a minimum of 30 min resting at room temperature, samples were centrifuged (1000*xg*, 10 min) and serum collected before storage at −80°C. Serum samples were analyzed using flow cytometric bead-based assay Biolegend LEGENDplex kit according to manufacturer’s instructions and software.

#### Tissue processing, and phenotyping and sorting of T cell subsets

To remove blood-borne cells and ensure that only parenchymal cells were analyzed, mice were subjected to cardiac perfusion with PBS. Following perfusion, lungs and liver were excised and collected in HBSS (Monash BDI media facility). Following cardiac perfusion, lungs and liver were harvested. Lungs were finely minced and digested in lung digestion buffer containing collagenase I (ThermoFisher) and DNase I (Sigma Aldrich) for 30 min at 37 °C, 5% CO_2_. Digested tissue was passed through a 70 μm filter into a 50 mL tube, washed twice with cRPMI (4.5% essential supplement, 9% FCS, 86.5% RPMI (Gibco)), and subjected to density gradient separation by underlaying with 70% Percoll (Sigma Aldrich) followed by centrifugation at 800*g* for 20 min at room temperature, with minimum deceleration. The lymphocyte layer at the interface was collected, washed once with cRPMI, filtered through a 40 μm strainer, resuspended in 500 μL cRPMI, and counted prior to flow cytometry staining. Livers were dissociated through a 100 μm filter in HBSS (Monash BDI media facility), washed with PBS, and resuspended in 37.5% Percoll (Sigma Aldrich). Samples were centrifuged at 690 g for 12 min at room temperature, and lymphocytes were collected from the pellet. The cells were washed twice with PBS, subjected to RBC lysis using 1 mL RBC lysis buffer (Sigma Aldrich) for 2 min at room temperature, and quenched with HBSS. Following centrifugation, the pellet was resuspended in 500 μL cRPMI, counted, and plated for flow cytometry staining.

Tissues were processed and single cell suspensions of splenocytes were stained for phenotyping or cell sorting. Cells required for proliferation assays were additionally stained with Cell Trace Violet (CTV) as per manufacturer’s instructions prior to cell sorting. T_N_ cells were defined as CD44^LO^, T_VM_ cells were defined as CD44^HI^ CD49d^LO^ and T_MEM_ cells were defined as CD44^HI^ CD49d^HI^ within the Live/TCR^+^/CD8^+^ T cell population as previously described^4^^,^^27^ and with gating shown in Figure S1E. The purity of sorted T cell populations was routinely >85% (Figure S1F).

Cell counts were determined using Accucheck beads (Invitrogen) as follows. A fixed number (10,000) of Accuchek beads (Invitrogen) from 200,000 beads/mL stock, were added to samples before flow cytometric acquisition. Total lymphocyte counts were then computed based on the following equation: Cells/ml of sample = (cell events/bead events) × (bead concentration (beads/ml)/volume of beads added (mL)).

For intracellular staining of cytokines and cytosolic molecules, cells were stained with surface markers and fixable viability dye prior to resuspension in 100 μL of BD Cytofix/Cytoperm Buffer for 30 min at 4°C. Cells were washed twice in 1X BD Perm/Wash Buffer and stained with antibodies for intracellular markers (diluted at required concentration in 1X BD Perm/Wash Buffer) for 30 min at 4°C. Cells were washed twice with 1X Perm/Wash Buffer and once in FACS buffer (1X PBS with 1% bovine serum albumin (BSA) and 0.02% sodium azide). Cells were resuspended in 100-200 μL of FACS buffer prior to flow cytometric analysis. For analyses of cytokine production by *in vitro* stimulated cells, GolgiPlug was added 5 h prior to harvest.

For intranuclear markers, cells were stained with surface markers and fixable viability dye prior to resuspension in 100 μL of 1X FoxP3 Fixation/Permeabilization Solution (30 min at room temperature) and washed once in 1X Permeabilization Buffer. Antibodies for intranuclear markers were diluted at required concentration in 1X Permeabilization Buffer and cells were then stained for 30–60 min at room temperature. Cells were washed twice with 1X Permeabilization Buffer with a final wash in FACS buffer. Cells were resuspended in 100-200 μL of FACS buffer prior to flow cytometric analysis. All flow cytometric acquisition was performed on the LSR II, LSRFortessa X-20 or FACSymphony A3 (BD Biosciences) and all flow cytometric sorting was performed on the FACSAria and Influx (BD Biossciences) cell sorter instruments. Flow cytometric analyses were performed using FlowJo software (version 10) (BD Biosciences, Ashland, OR).

#### *In vitro* stimulation and proliferation assays

For bulk TCR proliferation, CTV-labelled sorted cells were stimulated on plates coated with polyclonal stimulation antibodies (anti-muCD3ε (10 μg/ml), anti-muCD8α (10 μg/ml) and anti-muCD11a (5 μg/ml)) for 3 days in complete RPMI containing recombinant human IL-2 (10U/ml) at 37°C and 5% CO_2_. Samples were harvested, stained with viability dye and analyzed using flow cytometry to assess CTV dilutions. For IL-15 proliferation assays, CTV-labelled sorted cells were stimulated with 100 ng/ml cIL-15 (complexed IL-15, as previously described^27^) for 5 days at 37°C and 5% CO_2_. Samples were harvested, stained with viability dye and analyzed using flow cytometry to assess CTV dilutions. For single-cell TCR proliferation, sorted single cells were sorted into 96-well plates coated with stimulation antibodies (anti-muCD3ε (10 μg/ml), anti-muCD8α (10 μg/ml) and anti-muCD11a (5 μg/ml)) in complete RPMI containing recombinant human IL-2 (10U/mL). Plates were incubated for 4 days at 37°C and 5% CO_2_ before colonies were manually counted from individual wells on an inverted microscope.

#### Phosphorylation staining

To analyze basal phosphorylation of p38, cells were stained with surface markers and fixable viability dye. Cells were fixed with 1X Lyse/Fix solution at 37°C for 10 min, washed twice with ice-cold PBS and resuspended in ice-cold Perm Buffer II prior to overnight incubation at −20°C. Cells were washed twice with FACS buffer and stained with unconjugated phospho-p38 MAPK (Thr180/Tyr182) for 30 min at 4°C, washed twice with FACS buffer, and stained with PE-conjugated goat anti-rabbit IgG F(ab’)2 fragment for 30 min at room temperature. Cells were washed twice in FACS buffer prior to flow cytometric analysis. For analysis of phospho-ERK, cells were subjected to polyclonal stimulation for 0 to 12 min or left unstimulated. Cells were harvested at 5-, 10- and 12-min following stimulation and subjected to staining as above with unconjugated phospho-p44/42 MAPK (Erk1/2) (Thr202/Tyr204) and with PE-conjugated goat anti-rabbit IgG F(ab’)2 fragment.

### Quantification and statistical analysis

Statistical analyses were performed in GraphPad Prism (v9, v10). Statistical significance was determined using Mann-Whitney U nonparametric tests, with significance being defined as ∗ (*p* < 0.05) and ∗∗ (*p* < 0.01).

## Acknowledgments

The authors wish to thank Minh (Morgan) Bui for assistance with graphics and staffs at Monash Animal Research Platform and Monash Flowcore. This work was funded by the National Health and Medical Research Council (Australia) Investigator Grant to N.L.L.G. (2017335), and Australian Research Council Discovery Projects (DP20010277, DP230102412).

## Author contributions

N.L.L.G. and K.M.Q. conceived the study; N.L.L.G., T.H., K.L.G.-J., S.B., M.O.’K., and K.M.Q. designed experiments; N.L.L.G., T.H., and A.N. wrote the original manuscript; all co-authors provided feedback on the final manuscript. T.H., A.N., D.T., D.K., Z.H., E.S.P., A.K., and S.B. performed the experiments; T.H. and A.N. processed and analyzed the data.

## Declaration of interests

The authors declare no conflict of interest.

## References

[1] Miller C.H., Klawon D.E.J., Zeng S., Lee V., Socci N.D., Savage P.A. (2020). Eomes identifies thymic precursors of self-specific memory-phenotype CD8+ T cells. Nat. Immunol..

[2] Drobek A., Moudra A., Mueller D., Huranova M., Horkova V., Pribikova M., Ivanek R., Oberle S., Zehn D., McCoy K.D. (2018). Strong homeostatic TCR signals induce formation of self-tolerant virtual memory CD8 T cells. EMBO J..

[3] Thiele D., La Gruta N.L., Nguyen A., Hussain T. (2020). Hiding in Plain Sight: Virtually Unrecognizable Memory Phenotype CD8+ T cells. Int. J. Mol. Sci..

[4] Quinn K.M., Fox A., Harland K.L., Russ B.E., Li J., Nguyen T.H.O., Loh L., Olshanksy M., Naeem H., Tsyganov K. (2018). Age-Related Decline in Primary CD8+ T Cell Responses Is Associated with the Development of Senescence in Virtual Memory CD8+ T Cells. Cell Rep..

[5] White J.T., Cross E.W., Burchill M.A., Danhorn T., McCarter M.D., Rosen H.R., O’Connor B., Kedl R.M. (2016). Virtual memory T cells develop and mediate bystander protective immunity in an IL-15-dependent manner. Nat. Commun..

[6] Jacomet F., Cayssials E., Basbous S., Levescot A., Piccirilli N., Desmier D., Robin A., Barra A., Giraud C., Guilhot F. (2015). Evidence for eomesodermin-expressing innate-like CD8(+) KIR/NKG2A(+) T cells in human adults and cord blood samples. Eur. J. Immunol..

[7] Choi S.J., Koh J.-Y., Rha M.-S., Seo I.-H., Lee H., Jeong S., Park S.-H., Shin E.-C. (2023). KIR+CD8+ and NKG2A+CD8+ T cells are distinct innate-like populations in humans. Cell Rep..

[8] Haluszczak C., Akue A.D., Hamilton S.E., Johnson L.D.S., Pujanauski L., Teodorovic L., Jameson S.C., Kedl R.M. (2009). The antigen-specific CD8+ T cell repertoire in unimmunized mice includes memory phenotype cells bearing markers of homeostatic expansion. J. Exp. Med..

[9] Martinet V., Tonon S., Torres D., Azouz A., Nguyen M., Kohler A., Flamand V., Mao C.-A., Klein W.H., Leo O. (2015). Type I interferons regulate eomesodermin expression and the development of unconventional memory CD8(+) T cells. Nat. Commun..

[10] Sosinowski T., White J.T., Cross E.W., Haluszczak C., Marrack P., Gapin L., Kedl R.M. (2013). CD8 + Dendritic Cell Trans Presentation of IL-15 to Naive CD8+ T Cells Produces Antigen-Inexperienced T Cells in the Periphery with Memory Phenotype and Function. J. Immunol..

[11] Watson N.B., Patel R.K., Kean C., Veazey J., Oyesola O.O., Laniewski N., Grenier J.K., Wang J., Tabilas C., Yee Mon K.J. (2024). The gene regulatory basis of bystander activation in CD8+ T cells. Sci. Immunol..

[12] Rolot M., Dougall A.M., Chetty A., Javaux J., Chen T., Xiao X., Machiels B., Selkirk M.E., Maizels R.M., Hokke C. (2018). Helminth-induced IL-4 expands bystander memory CD8+ T cells for early control of viral infection. Nat. Commun..

[13] Chu T., Tyznik A.J., Roepke S., Berkley A.M., Woodward-Davis A., Pattacini L., Bevan M.J., Zehn D., Prlic M. (2013). Bystander-Activated Memory CD8 T Cells Control Early Pathogen Load in an Innate-like , NKG2D-dependent manner. Cell Rep..

[14] Murali-Krishna K., Altman J.D., Suresh M., Sourdive D.J., Zajac A.J., Miller J.D., Slansky J., Ahmed R. (1998). Counting antigen-specific CD8 T cells: a reevaluation of bystander activation during viral infection. Immunity.

[15] Quinn K.M., Hussain T., Kraus F., Formosa L.E., Lam W.K., Dagley M.J., Saunders E.C., Assmus L.M., Wynne-Jones E., Loh L. (2020). Metabolic characteristics of CD8+ T cell subsets in young and aged individuals are not predictive of functionality. Nat. Commun..

[16] Chiu B.-C., Martin B.E., Stolberg V.R., Chensue S.W. (2013). Cutting edge: Central memory CD8 T cells in aged mice are virtual memory cells. J. Immunol..

[17] Jin J.-H., Huang H.-H., Zhou M.-J., Li J., Hu W., Huang L., Xu Z., Tu B., Yang G., Shi M. (2020). Virtual memory CD8+ T cells restrain the viral reservoir in HIV-1-infected patients with antiretroviral therapy through derepressing KIR-mediated inhibition. Cell. Mol. Immunol..

[18] Lin J.S., Mohrs K., Szaba F.M., Kummer L.W., Leadbetter E.A., Mohrs M. (2018). Virtual memory CD8 T cells expanded by helminth infection confer broad protection against bacterial infection. Mucosal Immunol..

[19] Cayssials E., Jacomet F., Piccirilli N., Lefèvre L., Roy L., Guilhot F., Chomel J.-C., Leleu X., Gombert J.-M., Herbelin A., Barbarin A. (2019). Sustained treatment-free remission in chronic myeloid leukaemia is associated with an increased frequency of innate CD8(+) T-cells. Br. J. Haematol..

[20] Jacomet F., Cayssials E., Barbarin A., Desmier D., Basbous S., Lefèvre L., Levescot A., Robin A., Piccirilli N., Giraud C. (2016). The hypothesis of the human iNKT/innate CD8(+) T-cell axis applied to cancer: Evidence for a deficiency in chronic myeloid leukemia. Front. Immunol..

[21] Wang X., Waschke B.C., Woolaver R.A., Chen S.M.Y., Chen Z., Wang J.H. (2021). MHC class I-independent activation of virtual memory CD8 T cells induced by chemotherapeutic agent-treated cancer cells. Cell. Mol. Immunol..

[22] Smith T.R.F., Kumar V. (2008). Revival of CD8+ Treg-mediated suppression. Trends Immunol..

[23] Akane K., Kojima S., Mak T.W., Shiku H., Suzuki H. (2016). CD8+CD122+CD49dlow regulatory T cells maintain T-cell homeostasis by killing activated T cells via Fas/FasL-mediated cytotoxicity. Proc. Natl. Acad. Sci. USA.

[24] Dai H., Wan N., Zhang S., Moore Y., Wan F., Dai Z. (2010). Cutting edge: programmed death-1 defines CD8+CD122+ T cells as regulatory versus memory T cells. J. Immunol..

[25] Li J., Zaslavsky M., Su Y., Guo J., Sikora M.J., van Unen V., Christophersen A., Chiou S.-H., Chen L., Li J. (2022). KIR+CD8+ T cells suppress pathogenic T cells and are active in autoimmune diseases and COVID-19. Science.

[26] Lanzer K.G., Cookenham T., Reiley W.W., Blackman M.A. (2018). Virtual memory cells make a major contribution to the response of aged influenza-naïve mice to influenza virus infection. Immun. Ageing.

[27] Hussain T., Nguyen A., Daunt C., Thiele D., Pang E.S., Li J., Zaini A., O’Keeffe M., Zaph C., Harris N.L. (2023). Helminth Infection-Induced Increase in Virtual Memory CD8 T Cells Is Transient, Driven by IL-15, and Absent in Aged Mice. J. Immunol..

[28] Moudra A., Niederlova V., Novotny J., Schmiedova L., Kubovciak J., Matejkova T., Drobek A., Pribikova M., Stopkova R., Cizkova D. (2021). Phenotypic and Clonal Stability of Antigen-Inexperienced Memory-like T Cells across the Genetic Background, Hygienic Status, and Aging. J. Immunol..

[29] Lehmann J., Bellmann S., Werner C., Schröder R., Schütze N., Alber G. (2001). IL-12p40-dependent agonistic effects on the development of protective innate and adaptive immunity against Salmonella enteritidis. J. Immunol..

[30] Mastroeni P., Harrison J.A., Robinson J.H., Clare S., Khan S., Maskell D.J., Dougan G., Hormaeche C.E. (1998). Interleukin-12 is required for control of the growth of attenuated aromatic-compound-dependent salmonellae in BALB/c mice: Role of gamma interferon and macrophage activation. Infect. Immun..

[31] Mizuno Y., Takada H., Nomura A., Jin C.-H., Hattori H., Ihara K., Aoki T., Eguchi K., Hara T. (2003). Th1 and Th1-inducing cytokines in Salmonella infection. Clin. Exp. Immunol..

[32] Castleman M.J., Dillon S.M., Thompson T.A., Santiago M.L., McCarter M.D., Barker E., Wilson C.C. (2021). Gut bacteria induce granzyme B expression in human colonic ILC3s in vitro in an IL-15-dependent manner. J. Immunol..

[33] Teijaro J.R., Ng C., Lee A.M., Sullivan B.M., Sheehan K.C.F., Welch M., Schreiber R.D., de la Torre J.C., Oldstone M.B.A. (2013). Persistent LCMV infection is controlled by blockade of type I interferon signaling. Science.

[34] Wu W., Metcalf J.P. (2020). The role of type I IFNs in influenza: Antiviral superheroes or immunopathogenic villains?. J. Innate Immun..

[35] Butz E.A., Bevan M.J. (1998). Massive Expansion of Antigen-Specific CD8 T Cells during an Acute Virus Infection. Immunity.

[36] Flynn K.J., Riberdy J.M., Christensen J.P., Altman J.D., Doherty P.C. (1999). In vivo proliferation of naïve and memory influenza-specific CD8(+) T cells. Proc. Natl. Acad. Sci. USA.

[37] Mintern J.D., Bedoui S., Davey G.M., Moffat J.M., Doherty P.C., Turner S.J. (2009). Transience of MHC Class I-restricted antigen presentation after influenza A virus infection. Proc. Natl. Acad. Sci. USA.

[38] Cerny A., Sutter S., Bazin H., Hengartner H., Zinkernagel R.M. (1988). Clearance of lymphocytic choriomeningitis virus in antibody- and B-cell-deprived mice. J. Virol..

[39] Cooper L., Xu H., Polmear J., Kealy L., Szeto C., Pang E.S., Gupta M., Kirn A., Taylor J.J., Jackson K.J.L. (2024). Type I interferons induce an epigenetically distinct memory B cell subset in chronic viral infection. Immunity.

[40] Kupz A., Bedoui S., Strugnell R.A. (2014). Cellular requirements for systemic control of Salmonella enterica serovar Typhimurium infections in mice. Infect. Immun..

[41] Kupz A., Scott T.A., Belz G.T., Andrews D.M., Greyer M., Lew A.M., Brooks A.G., Smyth M.J., Curtiss R., Bedoui S., Strugnell R.A. (2013). Contribution of Thy1+ NK cells to protective IFN-γ production during Salmonella typhimurium infections. Proc. Natl. Acad. Sci. USA.

[42] Kambayashi T., Assarsson E., Lukacher A.E., Ljunggren H.-G., Jensen P.E. (2003). Memory CD8+ T cells provide an early source of IFN-gamma. J. Immunol..

[43] Berg R.E., Cordes C.J., Forman J. (2002). Contribution of CD8+ T cells to innate immunity: IFN-gamma secretion induced by IL-12 and IL-18. Eur. J. Immunol..

[44] McNally J.M., Zarozinski C.C., Lin M.Y., Brehm M.A., Chen H.D., Welsh R.M. (2001). Attrition of bystander CD8 T cells during virus-induced T-cell and interferon responses. J. Virol..

[45] Bahl K., Kim S.-K., Calcagno C., Ghersi D., Puzone R., Celada F., Selin L.K., Welsh R.M. (2006). IFN-induced attrition of CD8 T cells in the presence or absence of cognate antigen during the early stages of viral infections. J. Immunol..

[46] Welsh R.M., Bahl K., Marshall H.D., Urban S.L. (2012). Type 1 interferons and antiviral CD8 T-cell responses. PLoS Pathog..

[47] Jiang J., Lau L.L., Shen H. (2003). Selective depletion of nonspecific T cells during the early stage of immune responses to infection. J. Immunol..

[48] Xu A., Bhanumathy K.K., Wu J., Ye Z., Freywald A., Leary S.C., Li R., Xiang J. (2016). IL-15 signaling promotes adoptive effector T-cell survival and memory formation in irradiation-induced lymphopenia. Cell Biosci..

[49] Smith N.L., Patel R.K., Reynaldi A., Grenier J.K., Wang J., Watson N.B., Nzingha K., Yee Mon K.J., Peng S.A., Grimson A. (2018). Developmental origin governs CD8+ T cell fate decisions during infection. Cell.

[50] Kim H.-J., Wang X., Radfar S., Sproule T.J., Roopenian D.C., Cantor H. (2011). CD8+ T regulatory cells express the Ly49 Class I MHC receptor and are defective in autoimmune prone B6-Yaa mice. Proc. Natl. Acad. Sci. USA.

[51] Coles M.C., McMahon C.W., Takizawa H., Raulet D.H. (2000). Memory CD8 T lymphocytes express inhibitory MHC-specific Ly49 receptors. Eur. J. Immunol..

[52] Darrah P.A., Patel D.T., De Luca P.M., Lindsay R.W.B., Davey D.F., Flynn B.J., Hoff S.T., Andersen P., Reed S.G., Morris S.L. (2007). Multifunctional TH1 cells define a correlate of vaccine-mediated protection against Leishmania major. Nat. Med..

[53] Moffat J.M., Handel A., Doherty P.C., Turner S.J., Thomas P.G., La Gruta N.L. (2010). Influenza Epitope-Specific CD8+ T Cell Avidity, but Not Cytokine Polyfunctionality, Can Be Determined by TCR Clonotype. J. Immunol..

[54] La Gruta N.L., Turner S.J., Doherty P.C. (2004). Hierarchies in cytokine expression profiles for acute and resolving influenza virus-specific CD8+ T cell responses: correlation of cytokine profile and TCR avidity. J. Immunol..

[55] Rubinstein M.P., Kovar M., Purton J.F., Cho J.-H., Boyman O., Surh C.D., Sprent J. (2006). Converting IL-15 to a superagonist by binding to soluble IL-15R{alpha}. Proc. Natl. Acad. Sci. USA.

[56] Nishimura H., Hiromatsu K., Kobayashi N., Grabstein K.H., Paxton R., Sugamura K., Bluestone J.A., Yoshikai Y. (1996). IL-15 is a novel growth factor for murine gamma delta T cells induced by Salmonella infection. J. Immunol..

[57] Hirose K., Nishimura H., Matsuguchi T., Yoshikai Y. (1999). Endogenous IL-15 might be responsible for early protection by natural killer cells against infection with an avirulent strain of Salmonella choleraesuis in mice. J. Leukoc. Biol..

[58] Carrero J.A., Calderon B., Unanue E.R. (2004). Type I interferon sensitizes lymphocytes to apoptosis and reduces resistance to Listeria infection. J. Exp. Med..

[59] Haque A., Best S.E., Ammerdorffer A., Desbarrieres L., de Oca M.M., Amante F.H., de Labastida Rivera F., Hertzog P., Boyle G.M., Hill G.R., Engwerda C.R. (2011). Type I interferons suppress CD4^+^ T-cell-dependent parasite control during blood-stage Plasmodium infection. Eur. J. Immunol..

[60] Peacock C.D., Kim S.-K., Welsh R.M. (2003). Attrition of virus-specific memory CD8+ T cells during reconstitution of lymphopenic environments. J. Immunol..

[61] Wong R.S.M., Wu A., To K.F., Lee N., Lam C.W.K., Wong C.K., Chan P.K.S., Ng M.H.L., Yu L.M., Hui D.S. (2003). Haematological manifestations in patients with severe acute respiratory syndrome: retrospective analysis. BMJ.

[62] Nabeshima S., Murata M., Kikuchi K., Ikematsu H., Kashiwagi S., Hayashi J. (2002). A reduction in the number of peripheral CD28+CD8+T cells in the acute phase of influenza. Clin. Exp. Immunol..

[63] Hou S., Shao T., Mao T., Shi J., Sun J., Mei M., Tan X., Qi H. (2021). Virtual memory T cells orchestrate extralymphoid responses conducive to resident memory. Sci. Immunol..

[64] Zhang J.B., Chaurasia P., Nguyen A., Huang Z., Nguyen T.T., Xu H., Tran M.T., Reid H.H., Jones C.M., Schattgen S.A. (2025). LCK-co-receptor association ensures T cell lineage fidelity and maximizes epitope-specific TCR diversity. Sci. Immunol..

[65] Pereira B.I., De Maeyer R.P.H., Covre L.P., Nehar-Belaid D., Lanna A., Ward S., Marches R., Chambers E.S., Gomes D.C.O., Riddell N.E. (2020). Sestrins induce natural killer function in senescent-like CD8+ T cells. Nat. Immunol..

[66] Desai S., Landay A. (2010). Early immune senescence in HIV disease. Curr. HIV/AIDS Rep..

[67] Roederer M., Dubs J.G., Anderson M.T., Raju P.A., Herzenberg L.A., Herzenberg L.A. (1995). CD8 naive T cell counts decrease progressively in HIV-infected adults. J. Clin. Investig..

[68] Chauvin M., Sauce D. (2022). Mechanisms of immune aging in HIV. Clin. Sci. (Lond.).

[69] Effros R.B., Allsopp R., Chiu C.P., Hausner M.A., Hirji K., Wang L., Harley C.B., Villeponteau B., West M.D., Giorgi J.V. (1996). Shortened telomeres in the expanded CD28-CD8+ cell subset in HIV disease implicate replicative senescence in HIV pathogenesis. AIDS.

[70] Booiman T., Wit F.W., Girigorie A.F., Maurer I., De Francesco D., Sabin C.A., Harskamp A.M., Prins M., Franceschi C., Deeks S.G. (2017). Terminal differentiation of T cells is strongly associated with CMV infection and increased in HIV-positive individuals on ART and lifestyle matched controls. PLoS One.

[71] Battegay M., Cooper S., Althage A., Bänziger J., Hengartner H., Zinkernagel R.M. (1991). Quantification of lymphocytic choriomeningitis virus with an immunological focus assay in 24- or 96-well plates. J. Virol. Methods.

[72] Turner S.J., Carbone F.R., Strugnell R.A. (1993). Salmonella typhimurium delta aroA delta aroD mutants expressing a foreign recombinant protein induce specific major histocompatibility complex class I-restricted cytotoxic T lymphocytes in mice. Infect. Immun..

[73] Di Pietro A., Polmear J., Cooper L., Damelang T., Hussain T., Hailes L., O’Donnell K., Udupa V., Mi T., Preston S. (2022). Targeting BMI-1 in B cells restores effective humoral immune responses and controls chronic viral infection. Nat. Immunol..

