# Supplementary material for: LCMV-mediated loss of virtual memory CD8 T cells yields a functionally enhanced T cell subset
Source: iScience. 2025 Oct 28;28(11):113893. doi: 10.1016/j.isci.2025.113893 (PMC12639556; doi:10.1016/j.isci.2025.113893)
Supplement: Document S1. Figures S1 and S2 [file mmc1.pdf]

iScience, Volume *28*

Supplemental information

LCMV-mediated loss of virtual memory CD8 T cells yields a functionally enhanced T cell subset

Tabinda Hussain, Angela Nguyen, Daniel Thiele, Dulakara Kannangara, Zijian Huang, Ee Shan Pang, Alana Kirn, Sammy Bedoui, Kim L. Good-Jacobson, Meredith O'Keeffe, Kylie M. Quinn, and Nicole L. La Gruta

Figure S1

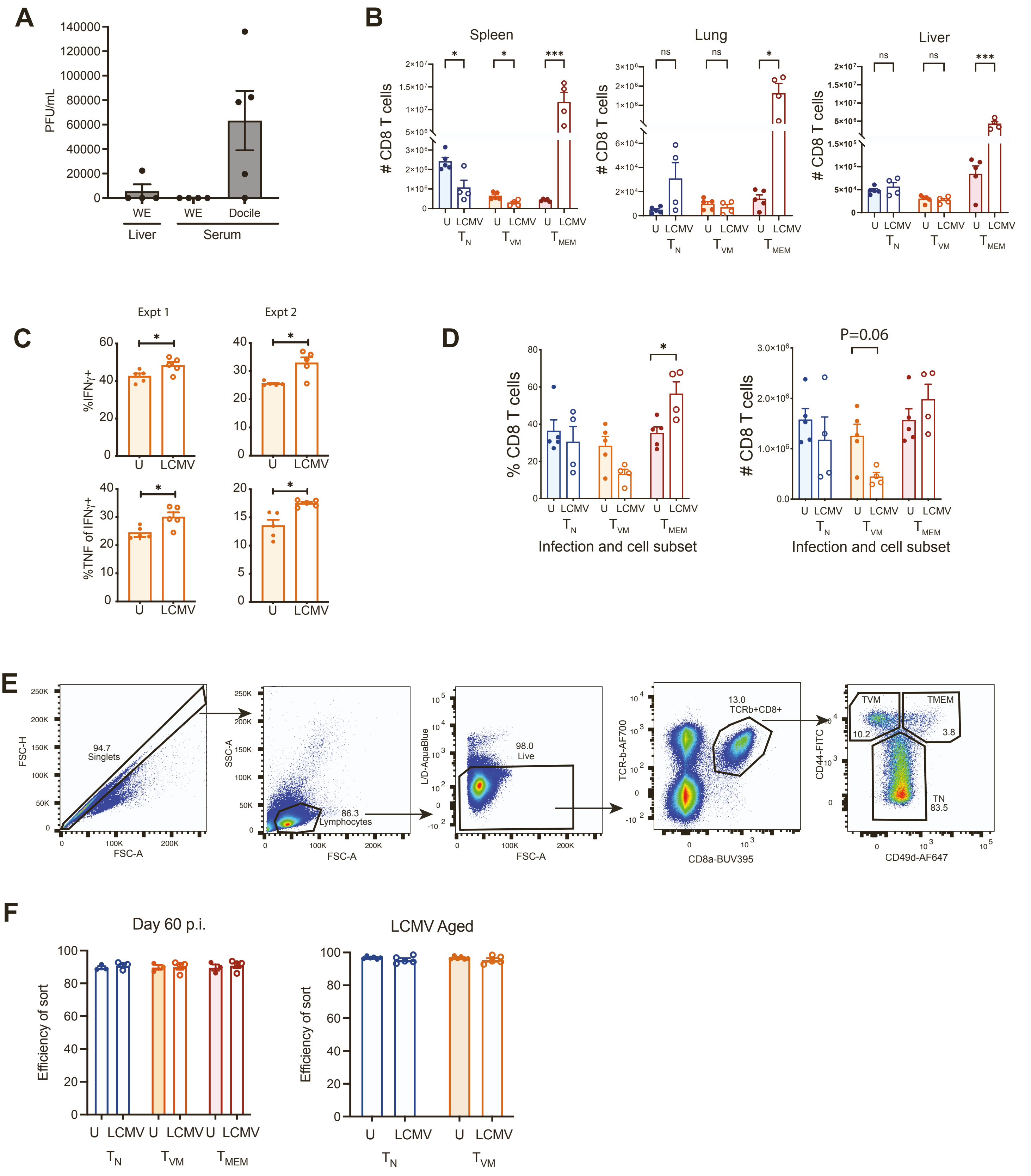

Figure S1. Viral clearance, T cell dynamics and phenotypic characterization of T cells after LCMV infection. A) Mice were infected with either 3000 pfu LCMV-WE strain (acute) or 2 x 10^6 pfu LCMV-Docile strain (chronic). Liver homogenates and serum were assessed for infectious virus using a focus formation assay at d14 after infection. **B**) Young B6 mice were infected with 3000 pfu LCMV-WE and spleen, lungs and livers harvested at d10 after infection for analysis of T_N, T_{VM} and T_{MEM} cell numbers. **C**) A breakdown of %IFN and %TNF of IFNγ+ from individual experiments shown combined in Figure 3C. **D**) A second experiment showing T_N, T_{VM} and T_{MEM} cells from LCMV-infected and then aged (LCMV) and uninfected (U) mice (left) and bar graph depicting frequency (left) and number (right) of cells in each subset. **E**) Gating strategy employed throughout the study. **F**) Efficiency of sorting for various T cell subsets based on sort reports from a BD Influx cell sorter.

Figure S2

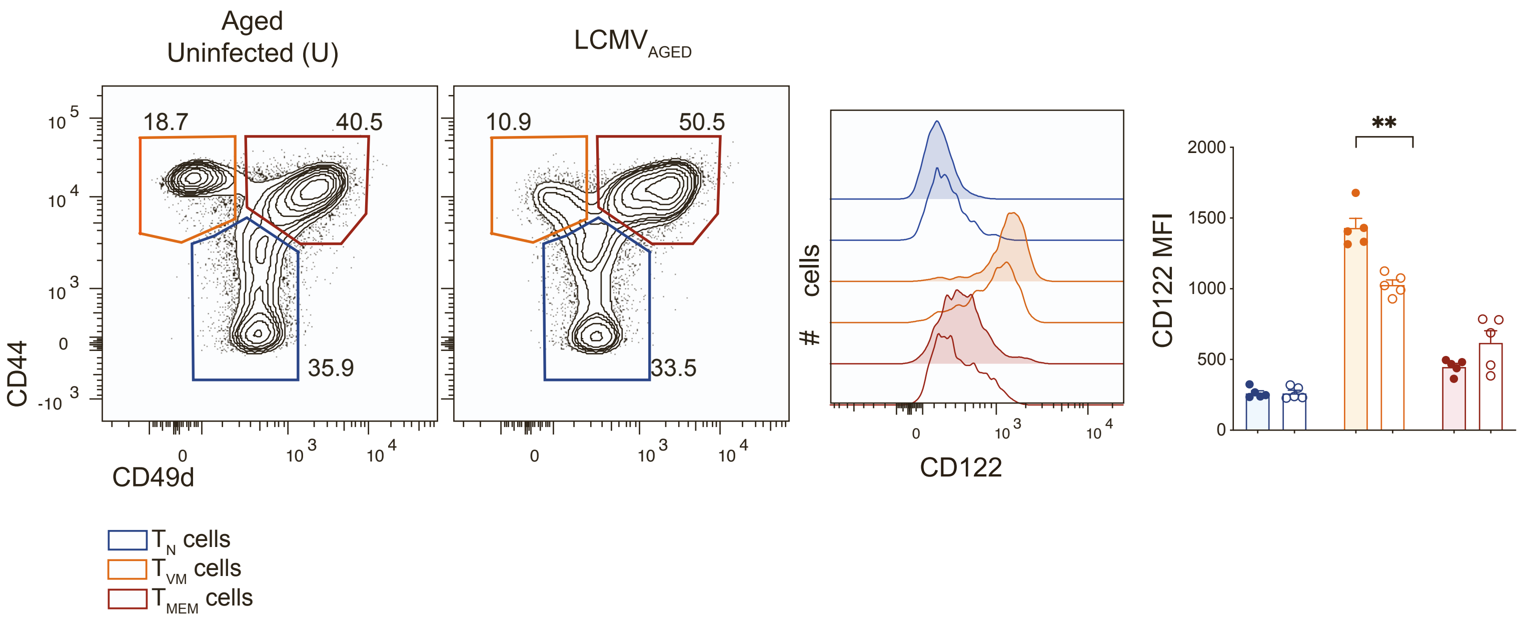

Figure S2. Aged cells classified as T_{VM} cells show sustained levels of CD122 expression. Young B6 mice were infected or not with LCMV and then aged to 18-20 mo. Representative contour plots gated on Live/TCR+/CD8+ T cells and depicting T_N, T_{VM} and T_{MEM} cells from LCMV-infected and then aged ($LCMV_{AGED}$) and uninfected (U) aged mice, representative histograms showing CD122 expression on cell subsets and bar graph depicting CD122 MFI for all cells.
